# Molecular Detection of *Histoplasma capsulatum* in Small Wild Mammals, Dogs, and Cats from Areas of Remaining Forest in the Brazilian Amazon

**DOI:** 10.1155/2023/5943212

**Published:** 2023-09-26

**Authors:** Diana Maria de Farias, Flávia de Nazaré Leite Barros, Francisco Dantas Sampaio-Júnior, Juliana dos Santos Cruz Vieira, Thamirys de Sousa Gonçalves, Amanda de Nazaré da Costa Rodrigues, Renata Cecília de Lima Macedo, Valíria Duarte Cerqueira, Ana Cristina Mendes de Oliveira, Giselle Souza da Paz, Gustavo Góes-Cavalcante, Alessandra Scofield

**Affiliations:** ^1^Laboratory of Animal Health, Institute of Veterinary Medicine, Federal University of Pará (UFPA), Castanhal, Brazil; ^2^Vertebrate Zoology Laboratory, Institute of Biological Sciences, Federal University of Pará (UFPA), Belém, Brazil; ^3^Laboratory of Animal Pathology, Institute of Veterinary Medicine, Federal University of Pará (UFPA), Castanhal, Brazil; ^4^Department of Veterinary Hygiene and Public Health, School of Veterinary Medicine and Animal Science, UNESP São Paulo State University, Botucatu, Brazil

## Abstract

*Histoplasma capsulatum* is the etiological agent of histoplasmosis, which can infect birds and different mammal species, including humans. In Brazil, the disease is not notifiable, and little is known about its infection in domestic and wild mammals. This study aimed to perform the molecular detection of *H. capsulatum* in small wild mammals from peri-urban forest remnants and in dogs and cats peri-domiciled in rural communities adjacent to these fragments in the Pará State, Brazilian Amazon. Samples of lung, liver, and skin were collected from free-living rats and marsupials captured in three peri-urban forest patches, as well as blood and skin from dogs and cats. *H. capsulatum* DNA was detected by nested PCR amplification, with products sequenced and subjected to phylogenetic analysis. *H. capsulatum* DNA was detected in 9.5% (12/126) of small wild mammals, with rats having a higher frequency of positive animals (25.6%; 10/39) when compared to marsupials (2.3%; 2/87) (*p*=0.0001). The frequencies of positive dogs and cats were 1.6% (2/121) and 5.5% (1/18), respectively. A higher frequency of infection by *H. capsulatum* was observed among small wild mammals when compared to dogs and cats (*p*=0.0143). In conclusion, *H. capsulatum* infection occurs in rats, marsupials, dogs, and cats in the Brazilian Amazon, with rats being important sentinels of the presence of this fungus in areas of remaining forest.

## 1. Introduction

*Histoplasma capsulatum* is a dimorphic fungus that can infect different species of mammals, including humans [1–7], and is rarely reported in birds (*Eclectus roratus*) [8]. This fungus is the etiologic agent of histoplasmosis and was originally classified based on its geographic distribution, morphology, and pathogenicity into three varieties: *H. capsulatum* var. *capsulatum*, *H. capsulatum* var. *duboisii*, and *H. capsulatum* var. *farciminosum* [9, 10]. The variety *capsulatum* causes classical histoplasmosis in humans, and its highest occurrence is in the Americas. The variety *duboisii* causes histoplasmosis in humans and non-human primates, particularly in Central and Western Africa [11, 12], while the variety *farciminosum* causes granulomatous alterations or ulcerated lesions in the skin and mucous membranes of dogs in Japan [13], and epizootic lymphangitis in equids in Europe, Africa, and Asia [9, 14]. Nowadays, phylogenetic studies have revealed extensive genetic diversity, and new phylogenetic species/lineages have been proposed in the *Histoplasma* complex [15–19].

The transmission of *H. capsulatum* occurs by inhalation of microconidia formed during the mycelial phase, which are found in soils contaminated by the feces of some species of bats and birds [8, 20, 21]. Moreover, transmission via organ transplantation from infected donors has also been reported in human patients [22].

According to the Pan American Health Organization, histoplasmosis is endemic in some regions of North, Central, and South America and can be the cause of 5%–15% of the deaths of AIDS patients per year in these regions [23]. In Brazil, histoplasmosis is endemic in some areas of the Northeast, Southeast, and South of the country [20, 24–27]. In 2011, the incidence of human histoplasmosis in the country was 2.19 per 1,000 hospital admissions, and this disease is the third leading cause of hospitalization due to mycotic infections [28]. Despite the importance of this disease for public health, it is not a notifiable disease; therefore, epidemiological data are scarce and the occurrence is underestimated in different Brazilian regions [26, 29–31]. In addition to human cases, histoplasmosis has been diagnosed in dogs [32], cats [33], equines [3], birds [8], and rabbits [4], while asymptomatic infection has been reported in bats, marsupials, rodents, xenarthras, and non-human primates [7, 11, 34, 35].

In dogs and cats, histoplasmosis is usually subclinical, and, in the case of clinical manifestations, signs are nonspecific, including fever, anorexia, progressive weight loss, and lethargy [5, 33]. Among wild animals, bats are important in the epidemiology of *H. capsulatum*, as they can be chronic carriers and disseminators of the agent, eliminating viable forms in their feces [7, 18, 35].

In Brazil, studies on *H. capsulatum* infection in wild and domestic animal species are still incipient, particularly in the Amazon. This region has one of the largest and most complex biomes in the world, which holds one of the greatest faunal diversities on the planet [36, 37]. However, for years, deforestation has led to forest fragmentation and the loss of habitat for wild animals [38, 39]. Therefore, this study aimed to perform the molecular detection of *H. capsulatum* in small wild mammals from peri-urban forest remnants and in dogs and cats peri-domiciled in rural communities adjacent to these fragments in the Pará State, Brazilian Amazon.

## 2. Material and Methods

### 2.1. Authorization for the Study

The study was approved by the Ethics Committee on Animal Use of the Evandro Chagas Institute (CEUA/IEC, protocol n°. 028/2014) and by the Biodiversity Authorization and Information System (SISBIO/ICMBIO, protocol n°. 37174-1).

### 2.2. Animals and Study Sites

The study was carried out with free-living rats and marsupials captured in three peri-urban forest remnants. Dogs and cats residing in rural communities adjacent to these forest remnants were also examined.

The forest fragments are located in the Expedito Ribeiro settlement (01°12ʹ54ʺS and 48°15ʹ55ʺW) in the city of Santa Bárbara do Pará, in the Centro Alegre community (01°11ʹ53.9ʺS and 046°18ʹ01.3ʺW) in the city of Viseu, and in the Ananin community (01°06ʹ29.1ʺS and 047°19ʹ52.9ʺW) in the city of Peixe-Boi, all in the state of Pará, Brazil. These three areas were selected based on their primary forest fragments with a medium to high level of degradation.

### 2.3. Biological Samples Collection

**Figure 1:**
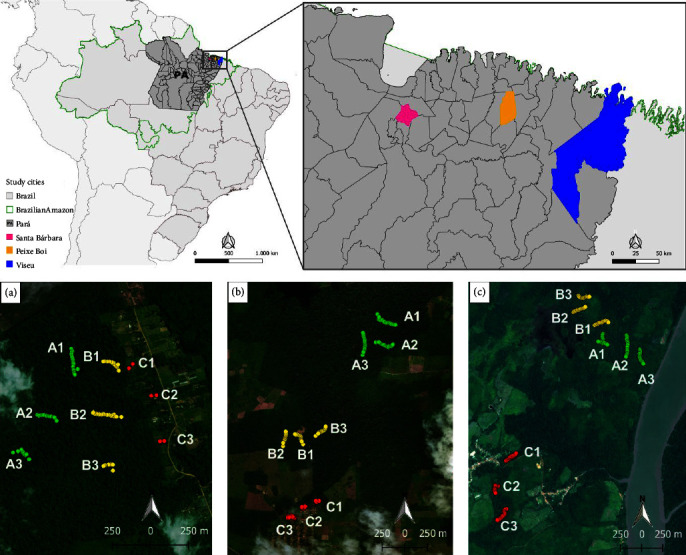
Geographic location of forest fragments and capture points of rats and marsupials in areas with different degrees of human interference (A, B, C) in the cities of Santa Bárbara do Pará (a), Peixe-Boi (b), and Viseu (c), State of Pará, Brazil.

From October 2014 to September 2015, five field expeditions were carried out, two in the municipality of Santa Bárbara do Pará, two in Peixe-Boi, and one in Viseu. Sampling was carried out within and close to the forest remnants. To capture wild rats and marsupials, 27 sample plots were established, nine in each forest fragment, divided into three different habitat types and defined as locations A, B, and C (Figure 1). The sites were distributed according to a distance gradient between the forest and peri-urban areas. Sites A were selected within the forest remnant, further away from the dwellings (Figure 1). In turn, sites B were positioned closer to the edge of the forest, near the dwellings. Finally, sites C were located outside the forest area, near the dwellings (Figure 1).

The small wild mammals were chemically restrained with an association of ketamine (10 mg/kg) and xylazine (1 mg/kg) intramuscularly. Then, all rat specimens and three specimens of each marsupial species were euthanized with an overdose of ketamine and xylazine, following the guidelines of the National Council for the Control of Animal Experimentation (CONCEA) and Resolution n°. 1000/12 of the Federal Council of Veterinary Medicine [40]. All specimens of small mammals were deposited in the Mastozoology Collection of the Federal University of Pará, Belém, Pará State, Brazil.

The animals were necropsied, and fragments of different tissues (liver, lung, and skin) were collected. Fragments were stored in 1.5 mL microtubes, identified, and kept in a freezer at −20°C for molecular analysis.

Skin samples were collected from captured and non-euthanized marsupials, and after anesthetic recovery, they were released at capture sites.

The species of rats and marsupials were identified by specialists in small Amazonian mammals at the Federal University of Pará, through morphological analyses, including body and cranial measurements.

For the study on dogs and cats, tutors had to sign an informed consent form to authorize the collection of whole blood and skin fragment samples. Moreover, a questionnaire was filled out to perform a general clinical examination, in which heart and respiratory rates, body temperature, mucosal color, and nutritional status were evaluated.

For the collection of biological samples, animals were physically and, when necessary, chemically restrained with 0.02% acepromazine (0.1 mL/kg) for dogs and with a combination of zolazepam hydrochloride and tiletamine hydrochloride (Zoletil 50™, Virbac, Brazil) (0.2 mL/kg) for cats. Whole blood samples were collected aseptically by puncture of the cephalic vein with scalp vein set 23G attached to a 5 mL syringe sterile and were stored in tubes containing ethylenediaminetetraacetic acid (EDTA). Skin biopsy was performed aseptically with sterile scissors and forceps, and tissue fragments were stored in 1.5 mL DNAse-free microtubes containing sterile saline. All samples were stored in a freezer at −20°C for further molecular analysis.

### 2.4. Molecular Analysis

DNA was extracted from whole blood samples of animals using the Illustra™ Blood Genomic Prep Mini Spin Kit (GE Healthcare), following the manufacturer's protocol. For DNA extraction from other tissue samples, the Illustra™ Tissue & Cells Genomic Prep Mini Spin Kit (GE Healthcare) was used, following the manufacturer's protocol with modifications, in which samples are incubated overnight in proteinase K.

To confirm the absence of PCR inhibitors in DNA samples, PCR was performed to detect the glyceraldehyde-3-phosphate dehydrogenase (GAPDH) gene, according to the protocol of Birkenheuer et al. [41], with modifications. The primers used were GAPDH F (AGCCTTCTCCATGGTGGTGAAGAC) and GAPDH R (CGGAGTCAACGGATTTGGTCG), which amplify a 437 base pair product of the GAPDH gene [41]. The amplification solution was composed of buffer (100 mM Tris-HCl, pH = 8.5; 500 mM KCl), 50 mM MgCl_2_, 2.0 units of Taq DNA polymerase (Ludwig Biotec™), 1 mM of each dNTP (dATP, dGTP, dCTP, and dTTP), 5 pmoles of each primer, and 5 *µ*L of test DNA, for a final volume of 25 *µ*L of solution. The temperature protocol was 94°C for 3 min, followed by 35 cycles of 94°C for 1 min, 56°C for 2 min, and 72°C for 2 min, with a final extension of 72°C for 7 min. The search for *H. capsulatum* DNA was performed only on PCR-positive samples for GAPDH.

Nested PCR was performed following the protocol of Bialek et al. [42], with modifications for the search for *H. capsulatum* DNA. In the first reaction, primers HCI (5ʹ-GCGTTCCGAGCCTTCCACCTCAAC-3ʹ) and HCII (5ʹ-ATGTCCCATCGGGCGCCGTGTAGT-3ʹ) were used, which amplify a fragment of 391 base pairs (bp). On the other hand, in the second reaction, primers used were HCIII (5ʹ-GAGATCTAGTCGCGGCCAGGTTCA-3ʹ) and HCIV (5ʹ-AGGAGAGAACTGTATCGGTGGCTTG-3ʹ) which amplify a 210 bp final product of the gene encoding the 100-kDa-like protein from *H. capsulatum* [42].

Buffer (100 mM Tris-HCl, pH = 8.5, 500 mM KCl), 50 mM MgCl_2_, 2.5 units of Taq DNA polymerase (Ludwig Biotec™), 100 mM of each dNTP (dATP, dGTP, dCTP, and dTTP), and 15 pmol of each primer were used for the amplification solution. In the first reaction, 5 *µ*L DNA (∼27.1 *η*g/*µ*L), and in the second reaction, 1 *µ*L of the product of the first reaction was used, totaling a final volume of 25 *µ*L.

Reactions were performed in a thermocycler (Veriti 96 Well Thermal Cycler–Applied Biosystems™). In the first reaction, a temperature protocol was used with an initial denaturation step at 94°C for 5 min, followed by 35 cycles of 94°C for 30 s, 50°C for 30 s, and 72°C for 1 min, with a final extension of 72°C for 5 min. In the second reaction, the temperature protocol used had an initial denaturation step at 94°C for 5 min, followed by 30 cycles of 94°C for 30 s, 65°C for 30 s, and 72°C for 1 min, with a final extension at 72°C for 5 min.

In all reaction batteries, positive control, negative control, and contamination control were used. The positive control was *H. capsulatum* DNA extracted from *H. capsulatum* isolate 144/15 culture from *Eptesicus brasiliensis*, provided by the University of São Paulo State (UNESP), Botucatu, São Paulo city, Brazil. Negative controls were samples of DNA extracted from the liver of *Rattus norvegicus* (Wistar) from the vivarium and DNA extracted from the blood of a *D. marsupialis* pup, a dog, and a cat negative in nested PCR for *H. capsulatum*. The contamination control was the amplification solution and double-distilled water without a DNA sample.

All reaction products were analyzed by electrophoresis in a 2% agarose gel and stained with GelRed™ (Biotium™) dye. Amplified products were estimated using a standard of 50 pairs of bases (50 Base-Pair-Ladder, Ludwig Biotec™), and visualization was performed in a transilluminator coupled to a photo-documentation system (Image Lab™ V. 5.2, Bio-Rad).

### 2.5. Sequencing and Phylogenetic Analysis

The amplified products were purified with a commercial kit (ExoSAP-IT™ PCR Product Cleanup Reagent-Applies Biosystems™) and sequenced in an automatic sequencer (ABI Prism 3500 Genetic Analyzer, Applies Biosystems™), according to the ABI PRISM Big Dye Terminator sequencing protocol.

The consensus sequences were submitted to BLASTn (https://www.ncbi.nlm.nih.gov) to determine the identity of the sequences obtained in relation to the *H. capsulatum* sequences stored in GenBank. For sequence editing, the Geneious v.10.0.6 program [43] was used, and for the most similar sequences, the CD-HIT program [44] was used, totaling the 25 most representative strains. Subsequently, the nucleotide sequences were aligned in the Mafft v.7 program [45] implemented within the Aliview v.1.17.1 program [46].

To calculate the evolutionary models best suited to the data, the statistics were measured by the Jmodeltest v.2.0 program [47, 48]. Each of the groups was organized following the phylogenetic classification indicated by Sepúlveda et al. [17] and Moreira et al. [21].

The genetic similarity was calculated using the Kimura two-parameter model using MEGA 6 software [49]. Multiple alignments were performed, and a phylogenetic tree was constructed with maximum likelihood using the IQTree v.1.3.0 program [50] by Ultrafast Bootstrap method (UFboot) [51], both performed with 1,000 bootstrap replicates. Statistical values with supports lower than 70% were ignored. For a better phylogenetic resolution, we used *XM_045419905.1- Blastomyces dermatitidis* e *XM_002628281.2- Blastomyces gilchristii* as outgroup [52].

### 2.6. Statistical Analysis

To evaluate the association of *H. capsulatum* DNA detection frequency between captured rats and marsupials and between small wild mammals and dogs and cats, Fisher's exact test was used for independent samples, considering the value of *p* < 0.05. All analyses were performed using BioEstat™ version 5.0 software.

## 3. Results

*H. capsulatum* DNA was detected in 9.5% (12/126) of small wild mammals, more frequent in rats (25.6%; 10/39) than in marsupials (2.3%; 2/87), with a value of *p*=0.0001. These animals were positive in tissue samples from the liver (rats *n* = 6; marsupial *n* = 1), lung (rats *n* = 3; marsupial *n* = 1), and skin (rat *n* = 1). In addition, the frequency of positive dogs and cats was 2.2% (3/139), with 1.6% (2/121) of dogs (blood *n* = 1; skin *n* = 1) and 5.5% (1/18) of cats (skin *n* = 1).

A higher frequency of infection by *H. capsulatum* was observed among small wild mammals when compared to dogs and cats (*p*=0.0143).

The presence of positive small wild mammals was observed in forest fragments of the three areas of Santa Bárbara do Pará (*n* = 4) and Peixe-Boi (*n* = 4) and in two areas (B and C) of Viseu (*n* = 3) (Table 1), while the positive dogs and cats were from Peixe-Boi.

Wild mammals did not show signs suggestive of histoplasmosis, but a blood-positive dog showed clinical signs such as weight loss, loss of appetite, anorexia, lethargy, and generalized alopecia.

**Figure 2:**
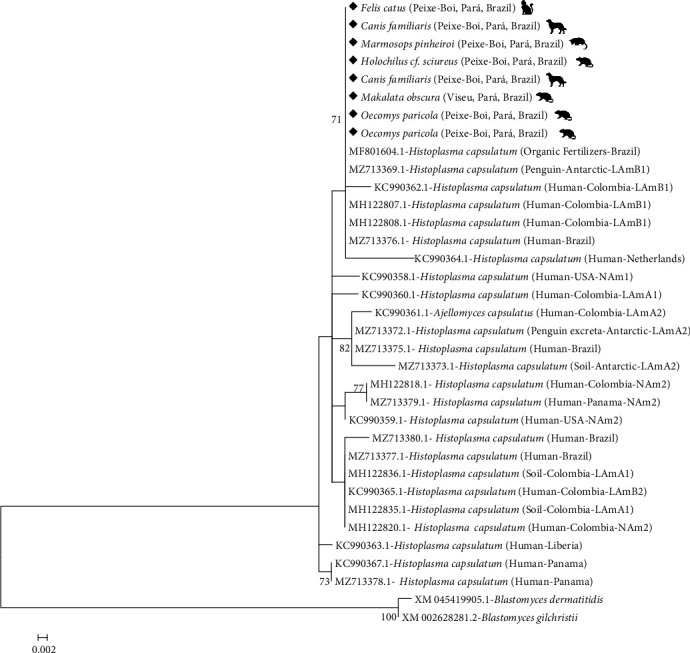
Phylogenetic tree built using the maximum likelihood with eight partial sequences of the gene encoding 100-kDa-like of *H. capsulatum* isolates in wild and domestic animals (highlighted) from the Brazilian Amazon and 25 sequences of *H. capsulatum* isolates deposited in GenBank (accession numbers shown). The bootstrap percentage of trees in which the associated taxa clustered together is shown next to the branches. The scale bar indicates the number of substitutions per site. The sequences from *Blastomyces dermatitidis* and *Blastomyces gilchristii* were used as an outgroup.

Blastn analysis revealed that the partial sequences of the gene encoding the 100-kDa-like protein of *H. capsulatum* from four isolates detected in rats, one isolate from a marsupial, two isolates from dogs, and one from a cat showed 100% identity with sequences of *H. capsulatum*, available from GenBank (MF801604.1, KX823346.1, MZ713371.1, MZ713370.1, MZ713369.1, KC990362.1). Phylogenetic analysis confirmed the eight sequence identities of *H. capsulatum* obtained in the present study, and these sequences were grouped in a clade with strains of the Latin American lineage (LAm), indicating a cohesive LAm B1 group with an internal bootstrap of 71%. A sequence from *Blastomyces dermatitidis* (XM045419905.1) and a sequence from *Blastomyces gilchristii* (XM002628281.2) were used as an outgroup (Figure 2).

## 4. Discussion

This study is the first report of infection by *H. capsulatum* in six species of rats (*H. megacephalus*, *H*. cf. *sciureus*, *M. obscura*, *Neacomys* sp. nov., *O. paricola*, and *R. rattus*), two species of marsupials (*M. murina* and *M*. cf. *pinheiroi*), and dogs and cats in the Brazilian Amazon.

Little information is available about *H. capsulatum* infection in humans and other animal species in the Brazilian Amazon. For more than 25 years, infections in mammals of the orders Xenarthra [53, 54], Rodentia, Marsupialia [34, 53, 55], and recently in bats captured in urban areas and a forest fragment in this region have been reported [7]. In the orders Rodentia and Marsupialia, *H. capsulatum* infection was diagnosed from isolated liver and spleen samples only in pacas (*Agouti paca*), agoutis (*Dasyprocta agouti* and *Myoprocta acouchy*), porcupines (*Coendou* sp.), and common skunk (*D. marsupialis*) [34].

In Brazil, *H. capsulatum* infection has been most often studied in bats, with rates ranging from 2.0% to 34.8% by molecular diagnosis [7, 56–58]. Nevertheless, few studies have been carried out with populations of free-living rats and marsupials [34, 53, 55, 59]. In the state of Pará, the frequency of infection by *H. capsulatum* (9.5%) observed in small wild mammals was higher than that obtained by Zancopé-Oliveira and Wanke [59] in rats and marsupials captured in areas with anthropic action in Rio de Janeiro State. These authors detected 3% (3/100) of infected animals through the isolation of *H. capsulatum* in the liver and/or spleen fragments. Isolation in culture and histopathology with the demonstration of *H. capsulatum* yeasts in tissues are the “gold standard” methods for the diagnosis of human histoplasmosis, especially in the disseminated form [60]. However, molecular methods have shown greater sensitivity for the detection of this agent when compared to other techniques [60, 61]. This may explain the higher frequencies in the Amazon biome, since nested PCR was used to detect *H. capsulatum* DNA in different biological samples from rats of the families Echimiidae, Cricetidae, and Muridae and marsupials of the family Didelphidae.

The infectious mycelial form of *H. capsulatum* develops preferentially in moist soils with high nitrogen contents and average temperatures of 25°C [62], characteristics similar to those found in the capture sites of small wild mammals in the present study. These animals showed a higher frequency of infection when compared to dogs and cats. Among small wild mammals, the detection of *H. capsulatum* DNA was more frequent in rats. Environmental characteristics added to the presence of contaminated soils and the terrestrial and/or arboreal habits of small wild mammals that shelter in tree hollows and soil nests may have favored the transmission of *H. capsulatum* to rats and marsupials in forest fragments [63]. Although the dispersion of *H. capsulatum* by these small wild mammals has not been proven, the data obtained corroborate studies by Zancopé-Oliveira and Wanke [59], who considered rats and marsupials as geographic markers for human histoplasmosis.

During expeditions, we observed that dogs and cats were kept in the peridomicile and had free access to different forest fragments in the three cities visited. However, in the city of Peixe-Boi, active deforestation was observed, and the suspension of infective fungal particles may have favored exposure of dogs and cats to infectious forms of *H. capsulatum*, as outbreaks of histoplasmosis in dogs and humans have already been reported after visits to areas with tree falls [64, 65].

As in human patients, dogs and cats infected with *H. capsulatum* may be asymptomatic or present with the respiratory and disseminated forms of histoplasmosis [6, 66]. In this study, one positive dog in a blood sample showed clinical signs suggestive of the disseminated form of histoplasmosis and was negative in molecular analyses for DNA detection of *Leishmania* spp., *Trypanosoma cruzi*, *Babesia vogeli*, *Ehrlichia canis*, and *Mycoplasma* spp. (unpublished data). It is important to emphasize that the clinical signs of canine histoplasmosis are generally nonspecific, and investigations of infections by other etiological agents are necessary for the differential diagnosis. In addition, one dog and one cat positive in skin samples were asymptomatic. According to data on feline histoplasmosis in the United States, this is the second most frequent systemic mycosis in the small animal clinic, with cats affected by skin lesions with or without pulmonary involvement [67, 68]. However, further research is needed for a better understanding of the importance of this disease in the dogs and cats medical clinic in Brazil.

In the last few years, new phylogenetic species/lineages in the *Histoplasma* complex have been described, and isolates from Latin America have contributed to the highest genetic diversity compared with other regions of the world [12, 16–18]. This diversity could be attributable to geological events, the existence of genetically distinct geographic populations of *Histoplasma*, and the differential dispersion potential of infected species of bats and other mammals [12, 15–17]. In the present study, the eight sequences of *H. capsulatum* showed a close association with the phylogenetic species Latin American group B1 (LAm B1) from Colombia and Antartica isolates. However, further regional epidemiological studies are needed to understand the diversity of phylogenetic species/lineages of the *Histoplasma* complex in the Brazilian Amazon and to assess possible risk factors for the occurrence of histoplasmosis in people and wild and domestic animals living in these areas, with the aim of constructing an efficient surveillance system for this etiologic agent that is so neglected in the country.

## 5. Conclusion

Infection by *H. capsulatum* occurs in small wild and domestic mammals in the Brazilian Amazon, with rats being important sentinels of the presence of this fungus in areas of peri-urban forest remnants.

## Figures and Tables

**Table 1:** Frequency of nested PCR-positive small wild mammals for *Histoplasma capsulatum* as a function of capture site in forest areas in the Brazilian Amazon.

Ordem/captured species	Frequency of positive animals by Nested-PCR for *Histoplasma capsulatum* per locations			Total
	A	B	C	
Rodentia				
* Echimys chrysurus*	0 (0/1)	n.c.	n.c.	0 (0/1)
* Hylaeamys megacephalus*	n.c.	0 (0/2)	50 (1/2)	25 (1/4)
* Holochilus* cf. *sciureus*	n.c.	n.c.	100 (1/1)	100 (1/1)
* Makalata obscura*	n.c.	100 (1/1)	n.c.	100 (1/1)
* Neacomys* sp. nov.	n.c.	n.c.	50 (1/2)	50 (1/2)
* Nectomys* cf. *rattus*	n.c.	n.c.	0 (0/1)	0 (0/1)
* Proechimys cuvieri*	0 (0/2)	0 (0/5)	0 (0/2)	0 (0/9)
* Oecomys* cf. *bicolor*	0 (0/1)	0 (0/2)	n.c.	0 (0/3)
* Oecomys* cf. *paricola*	37,5 (3/8)	100 (2/2)	n.c.	50 (5/10)
* Rattus rattus*	0 (0/2)	n.c.	20 (1/5)	14,28 (1/7)
Sub total	21,42 (3/14)	25 (3/12)	21,42 (4/13)	25,64% (10/39)
Didelphimorphia				
* Caluromys philander*	0 (0/2)	n.c.	n.c.	0 (0/2)
* Didelphis marsupialis*	0 (0/11)	0 (0/9)	0 (0/2)	0 (0/22)
* Gracilinanus* sp.	n.c.	n.c.	0 (0/3)	0 (0/3)
* Marmosa murina*	0 (0/6)	0 (0/15)	12,5 (1/8)	3,03 (1/29)
* Marmosa demerarae*	0 (0/4)	0 (0/4)	n.c	0 (0/8)
* Marmosops* cf. *pinheiroi*	0 (0/5)	20 (1/5)	0 (0/1)	9,09 (1/11)
* Metachirus* cf. *nudicaudatus*	n.c.	0 (0/2)	0 (0/5)	0 (0/7)
* Philander opossum*	n.c.	0 (0/2)	0 (0/3)	0 (0/5)
Sub total	0 (0/28)	2,7 (1/37)	4,54 (1/22)	2,3 (2/87)
Total	7,14 (3/42)	8,16 (4/49)	14,28 (5/35)	9,5 (12/126)

^*∗*^nc, non-captured.

## Data Availability

The data used to support the findings of this study are included within the article. The data that support the findings of this study are available from the corresponding author upon reasonable request.
